# Premenopausal serum androgens and breast cancer risk: a nested case-control study

**DOI:** 10.1186/bcr3117

**Published:** 2012-02-16

**Authors:** Anne Zeleniuch-Jacquotte, Yelena Afanasyeva, Rudolf Kaaks, Sabina Rinaldi, Stephanie Scarmo, Mengling Liu, Alan A Arslan, Paolo Toniolo, Roy E Shore, Karen L Koenig

**Affiliations:** 1 Department of Environmental Medicine, New York University School of Medicine, 650 First Avenue, New York, NY 10016, USA; 2 New York University Cancer Institute, New York University School of Medicine, 530 First Avenue, New York, NY 10016, USA; 3 Division of Cancer Epidemiology, German Cancer Research Centre, Im Neuenheimer Feld 280, D-69120 Heidelberg, Germany; 4 International Agency for Research on Cancer, 150, Cours Albert Thomas, 69372 Lyon Cedex 08, France; 5 Department of Obstetrics and Gynecology, New York University School of Medicine, 550 First Avenue, New York, NY 10016, USA; 6 Unit of Cancer Epidemiology, Institute of Social and Preventive Medicine, Centre Hospitalier Universitaire Vaudois, Biopôle 1, 2 Route de la Corniche, CH-1066 Epalinges, Switzerland; 7 Radiation Effects Research Foundation, 5-2 Hijiyama Park, Minami-ku, Hiroshima, 732-0815, Japan

## Abstract

**Introduction:**

Prospective epidemiologic studies have consistently shown that levels of circulating androgens in postmenopausal women are positively associated with breast cancer risk. However, data in premenopausal women are limited.

**Methods:**

A case-control study nested within the New York University Women's Health Study was conducted. A total of 356 cases (276 invasive and 80 *in situ*) and 683 individually-matched controls were included. Matching variables included age and date, phase, and day of menstrual cycle at blood donation. Testosterone, androstenedione, dehydroandrosterone sulfate (DHEAS) and sex hormone-binding globulin (SHBG) were measured using direct immunoassays. Free testosterone was calculated.

**Results:**

Premenopausal serum testosterone and free testosterone concentrations were positively associated with breast cancer risk. In models adjusted for known risk factors of breast cancer, the odds ratios for increasing quintiles of testosterone were 1.0 (reference), 1.5 (95% confidence interval (CI), 0.9 to 2.3), 1.2 (95% CI, 0.7 to 1.9), 1.4 (95% CI, 0.9 to 2.3) and 1.8 (95% CI, 1.1 to 2.9; *P*_trend _= 0.04), and for free testosterone were 1.0 (reference), 1.2 (95% CI, 0.7 to 1.8), 1.5 (95% CI, 0.9 to 2.3), 1.5 (95% CI, 0.9 to 2.3), and 1.8 (95% CI, 1.1 to 2.8, *P*_trend _= 0.01). A marginally significant positive association was observed with androstenedione (*P *= 0.07), but no association with DHEAS or SHBG. Results were consistent in analyses stratified by tumor type (invasive, *in situ*), estrogen receptor status, age at blood donation, and menopausal status at diagnosis. Intra-class correlation coefficients for samples collected from 0.8 to 5.3 years apart (median 2 years) in 138 cases and 268 controls were greater than 0.7 for all biomarkers except for androstenedione (0.57 in controls).

**Conclusions:**

Premenopausal concentrations of testosterone and free testosterone are associated with breast cancer risk. Testosterone and free testosterone measurements are also highly reliable (that is, a single measurement is reflective of a woman's average level over time). Results from other prospective studies are consistent with our results. The impact of including testosterone or free testosterone in breast cancer risk prediction models for women between the ages of 40 and 50 years should be assessed. Improving risk prediction models for this age group could help decision making regarding both screening and chemoprevention of breast cancer.

## Introduction

Prospective epidemiologic studies have consistently shown that circulating androgens in postmenopausal women are positively associated with breast cancer risk [1-8], an association which is thought to be largely due to their role as precursors of estrogens. Positive associations have also been reported for androgens in premenopausal women but data are limited to date, with the majority of studies having small numbers of cases [9-17]. If results in premenopausal women are confirmed, androgens could be considered for inclusion in breast cancer risk prediction models, such as the Gail model [18]. Improving breast cancer risk prediction models could be particularly valuable for women between the ages of 40 and 50, as they could help with decisions on screening, since guidelines for this age group are not consistent [19,20]. In addition, improved models might help younger women with an elevated risk of breast cancer decide whether to take tamoxifen for chemoprevention, as is recommended [21,22]. Tamoxifen has been approved by the US Food and Drug Administration for chemoprevention in women age 35 or older with a 5-year Gail-model risk greater than 1.66%, but is not often used in practice for this purpose [23,24]. A better understanding of the association between premenopausal concentrations of androgens and breast cancer risk is also important because some experimental studies [25,26], although not all [27], have suggested that androgens may protect against breast cell proliferation in an estrogen-rich environment, such as the time period before menopause. Finally, it is also important to assess the association between androgens and breast cancer risk because androgens have been advocated for relief of sexual symptoms such as low libido [28], including in older premenopausal women [29].

We report here the results of a case-control study nested within the New York University (NYU) Women's Health Study cohort. Prediagnostic concentrations of testosterone, androstenedione, dehydroepiandrosterone sulfate (DHEAS) and sex hormone-binding globulin (SHBG) were measured in serum samples from 356 incident cases and 683 controls who were premenopausal at enrollment (time of initial blood donation), and the association of these biomarkers with subsequent risk of breast cancer was assessed.

## Materials and methods

### Study Population

The NYU Women's Health Study (NYUWHS) enrolled 14,274 women 34 to 65 years old at a breast cancer screening center in New York City between 1985 and 1991 [30]. Women who had been pregnant or taken hormonal medications in the six months preceding their visit were excluded. After written informed consent was obtained, demographic, medical, anthropometric, reproductive, and dietary data were collected through self-administered questionnaires. Non-fasting peripheral venous blood was drawn prior to breast examination and serum samples were stored at -80°C for subsequent biochemical analyses. Up until 1991, women who returned to the clinic for annual breast cancer screening were asked to donate blood at each of their visits. For 52% of the women, two or more blood samples were collected.

Women were classified as premenopausal at enrollment/first blood donation if they reported at least one menstrual cycle in the six months prior to their visit. To determine the phase of cycle at blood donation, the start date of the menstrual period prior to the visit was recorded, and women were asked to return a postcard indicating the start date of their next menstrual cycle. Seventy-two percent of the women returned the postcard. For women with length of cycle 20 to 41 days, phase of cycle was calculated based on the number of days between the date of blood donation and the start date of the next menses: < 12 days: luteal; 12 to 16 days: peri-ovulatory; 17 to 19 days: late follicular; ≥20 days: early follicular. For the 15% of women who did not return the postcard but reported regular menstrual cycles, the date of menstrual period prior to the visit and the usual length of cycle were used to compute the phase of cycle at blood donation. Women who had had a hysterectomy without total oophorectomy prior to natural menopause and were less than 52 years of age at enrollment were also classified as premenopausal, with subsequent verification of menopausal status by follicle-stimulating hormone (FSH) measurements on a nested case-control basis. Women with a concentration < 12.75 mIU/mL were confirmed as premenopausal. For these women (7%), the phase of cycle at blood donation was coded as unknown. Phase of cycle was also coded as unknown for women who did not return the postcard and reported having irregular cycles and for women who returned the postcard but had a length of cycle less than 20 or more than 41 days (13%). The study was approved by the Institutional Review Board at the New York University School of Medicine.

### Nested case-control study of breast cancer

Breast cancer cases were identified through active follow-up of the cohort by mailed questionnaires approximately every two to four years and telephone interviews for non-respondents, as well as record linkages with state cancer registries in New York, New Jersey, and Florida, and with the US National Death Index. A capture-recapture analysis estimated the breast cancer case ascertainment rate in our cohort to be 95% [31]. Only incident cases (that is, diagnosed at least six months after blood donation) were included. Medical and pathology reports were reviewed to confirm the diagnosis.

For each case, two controls were selected at random from the appropriate risk set. The risk set for a case consisted of all women premenopausal at enrollment who were alive and free of cancer at the time of diagnosis of the case (index date) and who matched the case on age at enrollment/first blood donation (± 6 months), date of enrollment (± 3 months), number (0, 1, 2+) and dates (± 6 months) of subsequent blood donations, and phase (early follicular, late follicular, peri-ovulatory, luteal, unknown) and day of menstrual cycle at the first blood donation.

### Laboratory analyses

All assays were conducted in the Hormones and Cancer Group at the International Agency for Research on Cancer in Lyon, France. Assays were selected based on the results of a validity study [32]. Testosterone and DHEAS were measured by direct radioimmunoassays from Immunotech (Marseille, France), androstenedione and FSH by direct double-antibody radioimmunoassays from DSL (Diagnostic System Laboratories, Webster, Texas), and SHBG by a direct 'sandwich' immunoradiometric assay (Cis-Bio, Gif-sur-Yvette, France). Mean intra-batch and inter-batch coefficients of variation were 8.7% and 15.8% for testosterone, 7.8% and 13.5% for androstenedione, 5.4% and 14.7% for DHEAS and 5.6% and 13.5% for SHBG. Free testosterone was calculated using mass action equations and the concentrations of testosterone and SHBG [33].

### Statistical methods

Case and control characteristics were compared using the conditional logistic regression model, to take into account the individual matching. Median, 10^th ^and 90^th ^percentiles were calculated for the hormonal measurements and a mixed-effects regression model accounting for the matching was used to compare concentrations in cases and controls. Odds ratios were estimated using conditional logistic regression. Biomarkers were analyzed as quintiles based on the distribution of cases and controls combined, and trend tests were carried out using ordered categorical variables. We also conducted an analysis using the mean hormone level for women who had two samples, and the single available measurement for the remaining women. Finally, each biomarker was also analyzed after log_2_-transformation to estimate odds ratios corresponding to a doubling in concentration and to compute a trend test on the continuous scale. Adjusted models included known risk factors for breast cancer, that is, age at menarche, family history of breast cancer, parity, age at first birth, history of breast biopsy, and body mass index. Analyses were also done stratifying by tumor type (invasive and *in situ*), estrogen receptor status, age at enrollment, menopausal status at index date, lag time between blood donation and diagnosis of the case, and in the subgroup of women who had reported a history of regular menstrual cycles as well as five to seven cycles in the six months prior to blood donation. Analyses stratified by body mass index (BMI) and menopausal status at diagnosis and in the subgroup of women with five to seven regular menstrual cycles in the six months prior to blood donation were carried out using unconditional logistic regression, controlling for the matching factors, to avoid exclusion from the analysis of matched sets whose case and controls would be in different strata. All stratified analyses were carried out on log_2_-transformed hormones because of the smaller sample sizes in subgroups. The *P*-value obtained when adding a cross-product term to the model containing main effects was used to assess interaction. All statistical tests were two-sided.

The intra-class correlation coefficient (ICC) [34] was used to assess the temporal reliability of the biomarker measurements using two samples collected at different time points in cases and controls who donated blood more than once.

## Results

Among the 7,220 (50.6%) women who were premenopausal at the time of initial blood donation, 366 breast cancer cases (285 invasive and 81 *in situ*) were diagnosed by 1 January 2000. Ten cases were excluded because of FSH concentrations compatible with postmenopausal status. As a result, 356 cases (276 invasive and 80 *in situ*) are included in this analysis. Among the initially selected individually-matched controls, 29 were excluded because of their FSH concentrations, resulting in the inclusion of 683 controls in this analysis. For 138 cases and 268 controls, serum samples collected at two separate visits were analyzed.

Table 1 presents descriptive statistics of the study participants. Fifty-five percent of the cases were between the ages of 34 and 44 at blood donation. Fourteen percent of the cases were diagnosed before age 45 and 70% before age 55. As expected, there was a higher proportion of nulliparous women among cases than controls (40% versus 34%, *P *= 0.04) and cases tended to have a later age at first full-term pregnancy than controls (*P *= 0.03). There was also a higher proportion of women with a family history of breast cancer among cases than controls (*P *= 0.004). There was no difference between cases and controls in the proportions of overweight (25 to 29.9 kg/m^2^; 21% and 22%, respectively) and obese (≥30 kg/m^2^; 10% in both groups) women. Seventy-six percent of the cases and 78% of the controls had a history of regular cycles and also reported five to seven cycles in the six months prior to enrollment.

**Table 1:** Case and control subject characteristics

Characteristic	Case subjects (*n *= 356)	Control subjects (*n *= 683)	*P*-value^a^
Age at enrollment, years			matched
34 to 40	77 (22%)	152 (22%)	
40 to 44	118 (33%)	235 (35%)	
45 to 49	110 (31%)	205 (30%)	
≥50	51 (14%)	91 (13%)	
Age at diagnosis, years			
< 45	48 (14%)		
45 to 49	99 (28%)		
50 to 54	101 (28%)		
≥55	108 (30%)		
Menopausal status at index date			0.06^b^
Premenopausal	152 (49%)	316 (52%)	
Postmenopausal	161 (51%)	293(48%)	
Missing	43	74	
Age at menarche, years			0.49^c^
< 12	87 (25%)	157 (23%)	
12	97 (27%)	167 (25%)	
13	108 (30%)	210 (31%)	
> 13	64 (18%)	144 (21%)	
Missing	0	5	
Nulliparous (%)	142 (40%)	230 (34%)	0.04^b^
Age at first full-term pregnancy, years			0.03^c^
< 25	91 (44%)	238 (54%)	
25 to 30	54 (26%)	122 (27%)	
30 to 34	41 (20%)	60 (14%)	
≥35	21 (10%)	24 (5%)	
Missing	7	9	
First-degree family history of breast cancer (%)			0.004^b^
No	264 (74%)	550 (81%)	
Yes, one relative ≥45 years-old	64 (18%)	106 (15%)	
Yes, one relative < 45 years-old or more than one relative	28 (8%)	27 (4%)	
History of breast biopsy (%)	79 (22%)	127 (19%)	0.11^b^
Ever use of oral contraceptives (%)	175 (56%)	362 (59%)	0.37^b^
Missing	42	67	
Body mass index (kg/m^2^)			0.19^c^
< 20	55 (16%)	70 (10%)	
20 to 22.4	111 (31%)	233 (34%)	
22.5 to 24.9	79 (22%)	164 (24%)	
25 to 29.9	76 (21%)	149 (22%)	
≥30	34 (10%)	66 (10%)	
Missing	1	1	
Menstrual cycle regularity and number of periods in six months prior to blood donation			0.64^b^
Irregular cycles	78 (23%)	139 (21%)	
Regular cycles			
< 5 cycles in 6 months	3 (1%)	8 (1%)	
5 to7 cycles in 6 months	259 (76%)	516 (78%)	
> 7 cycles in 6 months	2	2	
Missing	14	18	

^a^Based on conditional logistic regression.^b^*P*-value for unordered categorical variable, except for family history of breast cancer (ordered categorical variable). ^c^*P*-value for variable on the continuous scale.

As was observed in longitudinal studies of premenopausal women [35], the highest concentrations of androgens were observed in the peri-ovulatory phase and the highest concentrations of SHBG in the luteal phase (data not shown). However, none of these differences were statistically significant. Table 2 shows hormone concentrations in cases and controls. Testosterone concentrations were higher in cases than controls (median: 0.90 nmol/L versus 0.83 nmol/L, *P *= 0.01) as were free testosterone concentrations (11.91 pmol/L versus 11.02 pmol/L, *P *= 0.01). The median androstenedione concentration was marginally higher in cases than controls (3.90 nmol/L versus 3.74 nmol/L, *P *= 0.08), whereas no statistically significant difference was observed for DHEAS and SHBG.

**Table 2:** Median (10^th ^and 90^th ^percentiles) of hormone concentrations in cases and controls

	Case subjects (*n *= 356)	Control subjects (*n *= 683)	*P*-value
Testosterone, nmol/L	0.90 (0.41, 2.07)	0.83 (0.31, 1.87)	0.01
Free testosterone, pmol/L	11.91 (4.92, 32.01)	11.02 (3.70, 27.81)	0.01
Androstenedione, nmol/L	3.90 (1.97, 6.80)	3.74 (1.80, 6.53)	0.08
DHEAS, μmol/L	3.58 (1.57, 7.22)	3.33 (1.54, 6.75)	0.50
SHBG, nmol/L	48.3 (22.3, 87.2)	49.4 (21.7, 89.5)	0.64

DHEAS, dehydroandrosterone sulfate; SHBG, sex hormone-binding globulin.

Table 3 reports odds ratios for breast cancer by quintiles of hormone concentrations. In multivariate-adjusted models, risk of breast cancer increased significantly with concentrations of testosterone (*P *= 0.04) and free testosterone (*P *= 0.01), with odds ratio (95% CI) of 1.8 (1.1, 2.9) and 1.8 (1.1, 2.8), respectively, for women in the highest versus lowest quintile. The increase in risk appeared more linear for free testosterone (odds ratios for quintiles were 1.0, 1.2, 1.5, 1.5 and 1.8) than testosterone (odds ratios for quintiles were 1.0, 1.5, 1.2, 1.4 and 1.8). A marginally significant trend (*P *= 0.07) of increasing risk with increasing concentration of androstenedione was also observed, while no significant association was observed between concentrations of DHEAS and SHBG with breast cancer risk. Associations were similar in analyses using the mean hormone level for women who had two samples and the single available measurement for the remaining women, except for testosterone for which higher odds ratios were observed when the average was used if available.

**Table 3:** Odds ratios (ORs) and 95% confidence intervals (CIs) for breast cancer by hormone concentration

	Quintiles					*P *for trend
		
	1	2	3	4	5	
Testosterone						
Cutpoints, nmol/L	< 0.50	0.50 to 0.74	0.75 to 0.99	1.00 to 1.41	> 1.41	
#cases/#controls^a^	58/139	75/124	63/134	70/129	80/118	
Unadjusted OR^b, c ^(95% CI)	1.0	1.5 (1.0, 2.3)	1.2 (0.8, 1.9)	1.4 (0.9, 2.2)	1.7 (1.1, 2.7)	0.06
Adjusted OR^b, c ^(95% CI)	1.0	1.5 (0.9, 2.3)	1.2 (0.7, 1.9)	1.4 (0.9, 2.3)	1.8 (1.1, 2.9)	0.04
Adjusted OR^b, c, d ^(95% CI)	1.0	1.6 (1.0, 2.6)	1.4 (0.9, 2.2)	1.6 (1.0, 2.6)	2.2 (1.3, 3.5)	0.03
Free Testosterone						
Cutpoints, pmol/L	< 6.02	6.02 to 9.11	9.12 to 13.91	13.92 to 20.92	> 20.92	
#cases/#controls^a^	60/136	63/134	72/125	69/128	81/116	
Unadjusted OR^b ^(95% CI)	1.0	1.1 (0.7, 1.7)	1.3 (0.9, 2.1)	1.3 (0.8, 2.0)	1.6 (1.0, 2.5)	0.03
Adjusted OR ^b, c ^(95% CI)	1.0	1.2 (0.7, 1.8)	1.5 (0.9, 2.3)	1.5 (0.9, 2.3)	1.8 (1.1, 2.8)	0.01
Adjusted OR^b, c, d ^(95% CI)	1.0	1.3 (0.8, 2.0)	1.5 (1.0, 2.4)	1.4 (0.9, 2.3)	1.9 (1.2, 2.9)	0.01
Androstenedione						
Cutpoints, nmol/L	< 2.45	2.45 to 3.37	3.38 to 4.32	4.33 to 5.58	> 5.58	
#cases/#controls^a^	65/138	72/120	63/139	70/132	80/123	
Unadjusted OR^b ^(95% CI)	1.0	1.2 (0.8, 1.9)	1.1 (0.7, 1.7)	1.2 (0.8, 1.9)	1.5 (1.0, 2.5)	0.11
Adjusted OR ^b, c ^(95% CI)	1.0	1.3 (0.8, 2.0)	1.1 (0.7, 1.7)	1.3 (0.8, 2.1)	1.7 (1.1, 2.7)	0.07
Adjusted OR^b, c, d ^(95% CI)	1.0	1.5 (0.9, 2.4)	1.0 (0.6, 1.6)	1.3 (0.8, 2.2)	1.6 (1.0, 2.7)	0.11
DHEAS						
Cutpoints, μmol/L	< 2.04	2.04 to 2.88	2.89 to 3.94	3.95 to 5.24	> 5.24	
#cases/#controls^a^	64/139	70/134	69/134	80/124	71/132	
Unadjusted OR^b ^(95% CI)	1.0	1.2 (0.8, 1.8)	1.2 (0.7, 1.8)	1.5 (0.9, 2.2)	1.2 (0.8, 1.9)	0.25
Adjusted OR ^b, c ^(95% CI)	1.0	1.1 (0.7, 1.7)	1.1 (0.7, 1.8)	1.5 (0.9, 2.3)	1.3 (0.8, 2.0)	0.14
Adjusted OR^b, c, d ^(95% CI)	1.0	1.1 (0.7, 1.8)	1.1 (0.7, 1.8)	1.3 (0.8, 2.0)	1.3 (0.8, 2.1)	0.20
SHBG						
Cutpoints, nmol/L	< 30.5	30.5 to 43.6	43.6 to 54.8	54.8 to 72.9	> 72.9	
#cases/#controls^a^	71/133	76/129	68/137	74/131	65/140	
Unadjusted OR^b ^(95% CI)	1.0	1.1 (0.8, 1.7)	0.9 (0.6, 1.4)	1.1 (0.7, 1.7)	0.9 (0.6, 1.3)	0.50
Adjusted OR ^b, c ^(95% CI)	1.0	1.1 (0.7, 1.7)	0.9 (0.6, 1.4)	1.1 (0.7, 1.5)	0.9 (0.6, 1.3)	0.37
Adjusted OR^b, c, d^(95% CI)	1.0	1.0 (0.6, 1.5)	0.9 (0.6, 1.4)	0.9 (0.6, 1.5)	0.7 (0.4, 1.2)	0.19

^a^The number of subjects varies for each hormone depending on the number of the values below the detection limit. ^b^Controlling for age, date, and phase and day of cycle at blood donation through matching and use of conditional logistic regression. ^c^Adjusted for age at menarche (< 12, 12, 13, > 13, missing), family history of breast cancer (no, one affected first-degree relative > 45 yrs old, one affected first degree relative < 45 yrs old or more than one affected first-degree relative), parity/age at first birth (≤20 years at first full-term pregnancy, 21-25 years at first full-term pregnancy, 26-30 years at first full-term pregnancy, > 30 years at first full-term pregnancy, nulliparous, missing), history of breast biopsy, and body mass index (< 20, 20-22.5, 22.6-24.9, 25-29.9, 30+, missing). ^d^Using the average of two measurements for women for whom two blood samples were available and one measurement for all other women and adjusting for all factors listed in ^c^. DHEAS, dehydroandrosterone sulfate; SHBG, sex hormone-binding globulin.

Table 4 reports odds ratios associated with a doubling of biomarker concentrations for all women, as well as by various subject characteristics. Although the odds ratios varied in magnitude according to subgroups and were not always consistently statistically significant, the associations between testosterone and free testosterone and breast cancer risk were usually in the same direction, and none of the tests for interaction was significant. In particular, odds ratios associated with a doubling in testosterone or free testosterone were elevated for invasive and *in situ *tumors, as well as for tumors diagnosed before and after menopause. Odds ratios greater than one were also observed for estrogen receptor-negative tumors, although the associations were weaker than for estrogen receptor-positive tumors and not statistically significant.

**Table 4:** Odds ratios (ORs) and 95% confidence intervals (CIs) for a doubling in hormone concentration for all women and according to subject characteristics^a^

Characteristic	Hormone				
(number of cases)	Testosterone	Free Testosterone	Androstenedione	DHEAS	SHBG
All women (*n *= 354)					
OR (95% CI)	1.6 (1.2, 2.3)	1.3 (1.1, 1.5)	1.3 (1.0, 1.7)	1.1 (0.9, 1.4)	0.9 (0.8, 1.1)
*P*-value	0.01	0.003	0.07	0.31	0.47
Tumor type					
Invasive (*n *= 274)					
OR (95% CI)	1.5 (1.1, 2.2)	1.2 (1.0, 1.4)	1.3 (0.9, 1.8)	1.1 (0.8, 1.4)	1.0 (0.8, 1.2)
*P*-value	0.03	0.02	0.11	0.60	0.84
In situ (*n *= 80)					
OR (95% CI)	2.3 (0.9, 5.9)	1.4 (1.0, 2.1)	2.0 (0.9, 4.5)	1.6 (0.9, 2.8)	0.8 (0.5, 1.3)
*P*-value	0.09	0.07	0.10	0.14	0.37
Estrogen receptor status^b^					
Positive (*n *= 104)					
OR (95% CI)	2.4 (1.2, 4.6)	1.6 (1.2, 2.2)	2.2 (1.3, 3.8)	1.2 (0.8, 1.8)	0.9 (0.6, 1.3)
*P*-value	0.01	0.003	0.01	0.42	0.56
Negative (*n *= 60)					
OR (95% CI)	1.7 (0.7, 4.3)	1.2 (0.9, 1.8)	0.7 (0.3, 1.6)	1.4 (0.7, 2.6)	0.8 (0.5, 1.4)
*P*-value	0.25	0.26	0.37	0.34	0.41
Age at enrollment^c^					
< 40 yrs (*n *= 77)					
OR (95% CI)	1.7 (0.7, 4.2)	1.3 (0.9, 2.0)	1.3 (0.6, 2.7)	0.9 (0.5, 1.5)	0.7 (0.4, 1.2)
*P*-value	0.27	0.19	0.45	0.61	0.17
40- to yrs (*n *= 117)					
OR (95% CI)	1.4 (0.7, 2.6)	1.1 (0.8, 1.4)	1.1 (0.7, 1.8)	1.1 (0.8, 1.6)	1.2 (0.8, 1.8)
*P*-value	0.33	0.50	0.72	0.53	0.36
≥45 yrs (*n *= 160)					
OR (95% CI)	1.9 (1.2, 3.2)	1.4 (1.1, 1.7)	1.5 (1.0, 2.3)	1.2 (0.9, 1.7)	0.9 (0.7, 1.2)
*P*-value	0.01	0.004	0.08	0.21	0.57
					
Menopausal status at diagnosis^d, e^					
Pre (*n *= 152)					
OR (95% CI)	1.4 (0.8, 2.3)	1.1 (0.9, 1.4)	1.0 (0.7, 1.5)	1.1 (0.8, 2.5)	1.0 (0.8, 1.4)
*P*-value	0.23	0.36	0.98	0.56	0.80
Post (*n *= 161)					
OR (95% CI)	1.7 (1.1, 2.6)	1.3 (1.1, 1.6)	1.3 (0.9, 1.9)	1.2 (0.8, 1.6)	0.9 (0.7, 1.1)
*P*-value	0.02	0.006	0.18	0.39	0.26
BMI^d^					
< 25 kg/m^2 ^(*n *= 239)					
OR (95% CI)	1.8 (1.2, 2.6)	1.3 (1.1, 1.5)	1.2 (0.9, 1.6)	1.3 (1.0, 1.6)	0.9 (0.7, 1.1)
*P*-value	0.003	0.003	0.30	0.08	0.41
25 to 29.9 kg/m^2 ^(*n *= 74)					
OR (95% CI)	2.4 (1.1, 5.0)	1.5 (1.1, 2.1)	1.9 (1.0, 3.5)	1.3 (0.8, 2.1)	1.1 (0.8, 1.7)
*P*-value	0.02	0.01	0.05	0.29	0.58
≥30 kg/m^2 ^(*n *= 108)					
OR (95% CI)	1.5 (0.8, 2.7)	1.2 (0.9, 1.6)	1.6 (1.0, 2.5)	1.0 (0.7, 1.5)	1.1 (0.8, 1.5)
*P*-value	0.20	0.11	0.08	0.95	0.64
Lag time between blood donation and diagnosis^f^					
< 7 yrs (*n *= 154)					
OR (95% CI)	2.0 (1.1, 3.5)	1.3 (1.0, 1.7)	1.3 (0.9, 2.1)	1.4 (1.0, 1.9)	1.0 (0.8, 1.5)
*P*-value	0.02	0.04	0.20	0.06	0.78
≥7 yrs (*n *= 200)					
OR (95% CI)	1.5 (1.0, 2.3)	1.3 (1.0, 1.5)	1.3 (0.9, 1.9)	1.0 (0.7, 1.3)	0.8 (0.6, 1.1)
*P*-value	0.07	0.02	0.17	0.80	0.22
5 to7 cycles in 6 months prior to enrollment and regular cycles^d ^(*n *= 253)					
OR (95% CI)	1.6 (1.1, 2.3)	1.2 (1.1, 1.5)	1.3 (1.0, 1.8)	1.1 (0.9, 1.5)	0.9 (0.8, 1.2)
*P*-value	0.02	0.01	0.07	0.29	0.59

^a^Adjusted for age at menarche (< 12, 12, 13, > 13, missing), family history of breast cancer (no, one affected first-degree relative > 45 yrs old, one affected first degree relative < 45 yrs old or more than one affected first-degree relative), parity/age at first birth (≤20 years at first full-term pregnancy, 21 to 25 years at first full-term pregnancy, 26 to 30 years at first full-term pregnancy, > 30 years at first full-term pregnancy, nulliparous, missing), history of breast biopsy, and body mass index (< 20, 20-22.5, 22.6-24.9, 25-29.9, 30+, missing). ^b^0.05 <*P*_interaction _< 0.15 for androstenedione. ^c^0.05 <*P*_interaction _< 0.15 for free testosterone. ^d^Using unconditional logistic regression, adjusting for matching factors in addition to factors listed in ^a^. ^e ^0.05 <*P*_interaction _< 0.15 for free testosterone. ^f^0.05 <*P*_interaction _< 0.15 for SHBG. DHEAS, dehydroandrosterone sulfate; SHBG, sex hormone-binding globulin.

Table 5 shows the ICCs for androgens and SHBG measured at two visits a median of two years (range 0.8 to 5.3 years) apart. ICCs were very similar in cases and controls. The lowest ICC was observed for androstenedione (0.57, 95% CI: 0.49, 0.65) with all other ICCs greater than 0.7.

**Table 5:** ICCs (95% CI) for hormonal biomarkers

	Cases *N *= 138	Controls *N *= 268
Testosterone	0.74 (0.65 - 0.81)	0.78 (0.73 - 0.82)
Androstenedione	0.58 (0.46 - 0.68)	0.57 (0.49 - 0.65)
DHEAS	0.82 (0.76 - 0.87)	0.76 (0.70 - 0.81)
SHBG	0.86 (0.81 - 0.90)	0.78 (0.73 - 0.82)
Free testosterone	0.86 (0.81 - 0.90)	0.82 (0.78 - 0.86)

DHEAS, dehydroandrosterone sulfate; SHBG, sex hormone-binding globulin.

## Discussion

We observed positive associations between premenopausal concentrations of total and free testosterone and breast cancer risk, with women in the highest quintile having a risk approximately 80% greater than women in the lowest quintile. We also observed a marginally significant positive association with androstenedione but no association with DHEAS or SHBG. The observation of similar associations (except for testosterone for which the odds ratios increased slightly) in an analysis using the mean hormone level for women who had two samples and the single available measurement for the remaining women strengthened our conclusions. There was no evidence of heterogeneity in the associations of total and free testosterone with breast cancer risk in subgroups according to tumor type, estrogen receptor status, age and BMI at enrollment, menopausal status at diagnosis and lag time between blood donation and diagnosis.

Two prospective studies also reported statistically significant positive associations between premenopausal concentrations of testosterone and free testosterone and breast cancer risk [14,17], three reported non-significant positive associations [10,12,16], and the smallest (17 cases) reported no association [9]. Overall, results are consistent across studies, despite variations in the phase of menstrual cycle when blood was drawn, follow-up duration, menopausal status at diagnosis and assay used (table 6). In particular, the two largest studies (the European Prospective Investigation into Cancer and Nutrition (EPIC) study and this one) both reported statistically significant positive trends for testosterone level and breast cancer risk; free testosterone was not evaluated in the EPIC study. We also observed a high temporal reliability of total testosterone and free testosterone over a median time of two years, as was also reported by others [36,37].

**Table 6:** Prospective studies of testosterone and breast cancer risk in premenopausal women^a^

Study	Number of Cases/Controls	Mean (SD) age at baseline for cases, yrs	Phase of cycle when blood was drawn	Mean follow-up duration, yrs	Menopausal status at diagnosis	Assay method	Testosterone Odds Ratio^b^ (95% CI)	*P*_trend_	Free Testosterone^c ^Odds Ratio^b^ (95% CI)	*P*_trend_
Guernsey (Thomas *et al. *1997)	62/182	40.9 (0.6)	Any	8.0 (range: < 1-16)	Not given	Direct radioimmunoassay	1.2 (0.6 to 2.4)	0.57	Not done	N/A
										
ORDET (Micheli *et al. *2004)	65/243	44.3 (4.9)	Luteal	5.2 (range: 3 to 8)	Not given	Direct radioimmunoassay	1.0 (ref) 1.1 (0.4 to3.0) 2.2 (0.6 to7.6)	0.28	1.0 (ref) 1.9 (0.6 to 5.8) 3.1 (0.9 to10.9)	0.08
										
EPIC (Kaaks *et al. *2005)	370/726	45.6 (7.6)	Any	2.8 (range: 0.2 to 5.8)	Not given Age < 49: 47%	Direct radioimmunoassay	1.0 (ref) 1.4 (1.0 tp 2.1) 1.4 (0.9 to 2.0) 1.7 (1.2 to 2.6)	0.01	Not done	N/A
										
NHS II (Eliassen *et al. *2006)	197/394	43.4 (3.8)	Follicular	2.9 (range: 0.1 to 7.3)	Pre^d^	Extraction, chromatography, radioimmunoassay	1.0 (ref) 1.3 (0.8 to 2.2) 1.4 (0.8 to 2.3) 1.3 (0.8 to 2.4)	0.35	1.0 (ref) 1.5 (0.8 to 2.6) 1.5 (0.9 to 2.6) 1.6 (0.9 to 2.8)	0.17
										
			Luteal				1.0 (ref) 1.3 (0.8 to 2.3) 1.4 (0.8 to 2.3) 1.6 (0.9 to 2.8)	0.10	1.0 (ref) 0.9 (0.5 to 1.5) 1.3 (0.7 to 2.2) 1.4 (0.8 to 2.5)	0.14
										
Columbia Serum Bank (Dorgan *et al. *2010)	98/168	44.7 (4.8)	Any	14.0 (SD: 6.6)	Not given Age ≥65: 68%	Extraction, chromatography, radioimmunoassay	1.0 (ref) 2.1 (0.9 to 4.8) 1.5 (0.6 to 3.4) 3.3 (1.5 to 7.5)	< 0.01	1.0 (ref) 1.7 (0.7 to 4.2) 1.7 (0.7 to 4.0) 4.2 (1.6 to 10.9)	< 0.01
										
NYUWHS	356/683	44.3 (4.9)	Any	13.1 (SD: 1.9)	Pre: 49% Post: 51%	Direct radioimmunoassay	1.0 (ref) 1.5 (0.9 to 2.3) 1.2 (0.7 to 1.9) 1.4 (0.9 to 2.3) 1.8 (1.1 to 2.9)	0.04	1.0 (ref) 1.2 (0.7 to 1.8) 1.5 (0.9 to 2.3) 1.5 (0.9 to 2.3) 1.8 (1.1 to 2.8)	0.01

^a^Wysowski *et al. *[9] not included in table because no odds ratio was provided (no statistical difference was observed in testosterone concentrations in 17 women). ^b^Odds Ratios (95% CIs) based on a 1 unit increase in the natural log of hormone concentration, tertiles, quartiles, or quintiles of serum concentration. ^c^Bioavailable testosterone (free plus albumin bound) calculated in Dorgan *et al. *2010 study. ^d^Except for five women.

It has been suggested that including circulating concentrations of sex hormones could improve risk prediction models [38,39]. In fact, imputed postmenopausal concentrations of estradiol improved the discriminatory accuracy of the log-incidence model for breast cancer risk prediction developed by Rosner and Colditz [40], although only modestly. Estrogens, though, are not good candidates for inclusion in risk prediction models of breast cancer in premenopausal women because no consistent association has been demonstrated with breast cancer risk in these women [9-11,14,16,17,41], which may be due to the large variations in estrogen concentrations over the menstrual cycle. The positive association of premenopausal testosterone and free testosterone concentrations with breast cancer risk, combined with the high temporal reliability of these biomarkers, suggests that it would be of interest to examine whether inclusion of one or the other could improve breast cancer risk prediction models. Testosterone was found to be associated with risk of breast cancer across strata of predicted risk (using either the Gail or the Rosner and Colditz models), suggesting that it may convey information about risk independent of the factors included in these models [42]. Free testosterone seems of particular interest for risk prediction models, because it is thought to be the fraction most readily available biologically, and because the association with risk appears more linear. Improving risk prediction models for younger women could have implications for both screening and chemoprevention decision making. For women between the ages of 40 and 49 years, recommendations for breast cancer screening are not consistent. Whereas the American Cancer Society and some professional societies continue to recommend annual mammography starting at age 40 [43], the US Preventive Services Task Force recommended in 2009 to start screening at age 50, rather than at age 40, in the absence of known underlying genetic mutation or history of chest radiation [19]. Regarding breast cancer prevention, there is a net benefit of tamoxifen for women below age 50 who have a Gail model five-year risk greater than 1.66% [44] and tamoxifen has been approved for chemoprevention in such women. Use of tamoxifen for prevention of breast cancer, though, has been limited [23,24]. It has been shown that the higher the risk of breast cancer relative to the risk of adverse events, the more likely a woman is to accept tamoxifen chemoprevention [45]. Factors helping to predict more accurately the absolute risk of breast cancer might thus lead to increased acceptance of chemoprevention by the women most likely to benefit and, therefore, result in a larger number of prevented breast cancers. A challenge that needs to be addressed prior to incorporation of circulating hormone levels in risk prediction models, though, is standardization of assay methods [38,46,47].

Positive associations between circulating androgens in postmenopausal women and risk of breast cancer have been observed consistently [1], although the association may vary according to the estrogen receptor (ER) status of the tumor. Whereas most studies found a positive association with ER-positive tumors [4,8,48,49], no significant association was observed in three studies [4,6,48], and the largest study to date found a significant inverse, rather than positive, association with ER-negative tumors [49]. Notwithstanding these differences, the main mechanism proposed to explain the increase in risk observed among postmenopausal women is the aromatization of androgens into estrogens in peripheral adipose tissue [50]. Because of the reduced production of estrogens by the ovaries, peripheral production is an important contributor to circulating concentrations of estrogens after menopause. This mechanism is thought to contribute to the well documented positive association between BMI and breast cancer risk observed after menopause [51]. It is unlikely, though, that this mechanism explains the association observed between androgens and breast cancer in premenopausal women because estrogens are mostly produced by the ovaries prior to menopause. Further, under this mechanism, one would expect a positive association between BMI and breast cancer risk in premenopausal women, as is seen in post-menopausal women, since more aromatization of androgens in adipose tissue is expected with increased BMI. However, although we did not observe an association of BMI with breast cancer risk in our study, an inverse, rather than positive, association between BMI and breast cancer risk in premenopausal women has been found in most studies [52,53]. Additional evidence against an important role of this mechanism is the observation made by Eliassen *et al. *that adjustment for concentrations of estradiol in premenopausal women did not affect risk estimates associated with testosterone concentrations [16]. It is of interest that we observed an association in women with regular cycles, as well as women of normal weight, suggesting that androgen concentrations increase breast cancer risk even in women with no evidence of hyperandrogenism.

In addition to their role as estrogen precursors, it has been proposed that androgens directly impact cell proliferation [54,55], possibly through binding to androgen receptors which are present in both normal breast tissue and most breast cancers [56]. Results from experimental studies, though, have been inconsistent, with some studies [25,26] reporting an inhibitory effect of androgens on estrogen-induced breast cell proliferation, while others did not [27]. The only human study that examined the effect of testosterone on breast cell proliferation found that postmenopausal women who received testosterone (300 μg/day patch) in addition to hormone replacement therapy (2 mg estradiol and 1 mg norethisterone acetate) did not have an increase in cell proliferation, while a more than five-fold increase in cell proliferation was observed in women who received only the estrogen + progestin therapy [57]. These studies, though, were conducted in primates or in women in the postmenopausal stage who received estrogens orally, the effect of which may differ from that of endogenous hormones. For instance, oral estrogens are known to increase production of SHBG and reduce the concentrations of free testosterone [58]. It is, therefore, not clear whether results of these studies apply to endogenous androgens in premenopausal women. Additional research is needed to explain why circulating concentrations of androgens in premenopausal women are associated with an increase in risk of breast cancer.

Androgens, in particular testosterone, have been proposed for relief of menopausal symptoms, in particular sexual desire deficit [28]. As symptoms may start well before menopause, androgen therapies may be prescribed beginning in the late premenopausal years [29,59]. In light of the increased risk of breast cancer associated with higher concentrations of circulating androgens both pre- and post-menopause, and the results of two prospective studies that reported an increased risk of breast cancer in women receiving estrogen + testosterone therapy [60,61], although this association was significant only in one of the two studies [60], caution should be exercised regarding long-term prescription of androgens.

The NYUWHS was designed primarily to examine the association of endogenous sex hormones with risk of breast cancer. We therefore excluded women taking exogenous estrogens and collected data on date of next menstrual period which allowed us to calculate the phase of cycle more precisely than some other studies. Other strengths of our study include the large number of cases, which allowed us to examine various subgroups and the availability of two serum samples in a fairly large number of both cases and controls which allowed us to show that a single androgen concentration measurement is quite representative of a woman's concentration over several years. This is despite the fact that we did not control for time of day of blood donation, and, therefore, for possible circadian variations of androgen production. A weakness of our study is that we used radioimmunassays without an extraction step, and the sensitivity and specificity of such assays have been questioned [46,62]. It should be noted, though, that results from other studies did not appear to vary according to whether or not a purification step was used (table 6). A similar observation was made for sex hormone concentrations in postmenopausal women [1].

## Conclusions

In conclusion, and in agreement with other cohorts, we observed associations between pre-diagnostic concentrations of total and free testosterone in premenopausal women and risk of breast cancer. These results suggest that androgen concentrations should be considered for inclusion in risk prediction models for women between the ages of 40 and 50, which could help in decision making regarding both screening and chemoprevention of breast cancer.

## Abbreviations

BMI: body mass index; CI: confidence interval; DHEAS: dehydroandrosterone sulfate; EPIC: European Prospective Investigation into Cancer and Nutrition; ER: estrogen receptor; FSH: follicle-stimulating hormone; ICC: intraclass correlation coefficient; NYUWHS: New York University Women's Health Study; SHBG: sex hormone-binding globulin.

## Competing interests

The authors declare that they have no competing interests.

## Authors' contributions

AZJ participated in the design of the study, statistical analysis, and manuscript preparation. YA performed the statistical analysis and contributed to manuscript preparation. RK participated in the conception of the study and manuscript preparation. SR performed serum hormone analyses and contributed to manuscript preparation. SS contributed to manuscript preparation. ML participated in the statistical analysis and manuscript preparation. AAA participated in data acquisition and manuscript preparation. PT and RES participated in the conception and design of the study and manuscript preparation. KLK participated in the design of the study, the statistical analysis and manuscript preparation. All authors read and approved the final manuscript.

## References

[B1] The Endogenous Hormones Breast Cancer Collaborative Group Endogenous sex hormones and breast cancer in postmenopausal women: reanalysis of nine prospective studies J Natl Cancer Inst 2002 94 606 616 10.1093/jnci/94.8.606 11959894

[B2] Kaaks R Rinaldi S Key TJ Berrino F Peeters PHM Biessy C Dossus L Lukanova A Bingham S Khaw KT Allen NE Bueno-de-Mesquita HB van Gils CH Grobbee D Boeing H Lahmann PH Nagel G Chang Claude J Clavel Chapelon F Fournier A Thibaut A Gonzlez CA Quirs JR Tormo MJ Ardanaz E Amiano P Krogh V Palli D Panico S Tumino R Postmenopausal serum androgens, oestrogens and breast cancer risk: the European prospective investigation into cancer and nutrition Endocr Relat Cancer 2005 12 1071 1082 10.1677/erc.1.01038 16322344

[B3] Woolcott C Shvetsov Y Stanczyk F Wilkens L White K Caberto C Henderson B Le-Marchand L Kolonel L Goodman M Plasma sex hormone concentrations and breast cancer risk in an ethnically diverse population of postmenopausal women: the Multiethnic Cohort Study Endocr Relat Cancer 2010 17 125 134 10.1677/ERC-09-0211 19903744 PMC2880171

[B4] Missmer S Eliassen AH Barbieri R Hankinson S Endogenous estrogen, androgen, and progesterone concentrations and breast cancer risk among postmenopausal women J Natl Cancer Inst 2004 96 1856 1865 10.1093/jnci/djh336 15601642

[B5] Zeleniuch-Jacquotte A Shore RE Koenig KL Akhmedkhanov A Afanasyeva Y Kato I Kim MY Rinaldi S Kaaks R Toniolo P Postmenopausal levels of estrogen, androgen, and SHBG and breast cancer risk: long-term results of a prospective study Br J Cancer 2004 90 153 159 10.1038/sj.bjc.6601517 14710223 PMC2395327

[B6] Baglietto L Severi G English D Krishnan K Hopper J McLean C Morris H Tilley W Giles G Circulating steroid hormone levels and risk of breast cancer for postmenopausal women Cancer Epidemiol Biomarkers Prev 2010 19 492 502 10.1158/1055-9965.EPI-09-0532 20086116

[B7] Manjer J Johansson R Berglund G Janzon L Kaaks R Agren A Lenner P Postmenopausal breast cancer risk in relation to sex steroid hormones, prolactin and SHBG (Sweden) Cancer Causes Control 2003 14 599 607 10.1023/A:1025671317220 14575357

[B8] Cummings S Lee J Lui L-Y Stone K Ljung B Cauleys J Sex hormones, risk factors, and risk of estrogen receptor-positive breast cancer in older women: a long-term prospective study Cancer Epidemiol Biomarkers Prev 2005 14 1047 1051 10.1158/1055-9965.EPI-04-0375 15894651

[B9] Wysowski DK Comstock GW Helsing KJ Lau HL Sex hormone levels in serum in relation to the development of breast cancer Am J Epidemiol 1987 125 791 799 3565354 10.1093/oxfordjournals.aje.a114596

[B10] Thomas H Key T Allen D Moore J Dowsett M Fentiman I Wang D A prospective study of endogenous serum hormone concentrations and breast cancer risk in premenopausal women on the island of Guernsey Br J Cancer 1997 75 1075 1079 10.1038/bjc.1997.183 9083346 PMC2222732

[B11] Kabuto M Akiba S Stevens R Neriishi K Land C A prospective study of estradiol and breast cancer in Japanese women Cancer Epidemiol Biomarkers Prev 2000 9 575 579 10868691

[B12] Micheli A Muti P Secreto G Krogh V Meneghini E Venturelli E Sieri S Pala V Berrino F Endogenous sex hormones and subsequent breast cancer in premenopausal women Int J Cancer 2004 112 312 318 10.1002/ijc.20403 15352045

[B13] Page J Colditz G Rifai N Barbieri R Willett W Hankinson S Plasma adrenal androgens and risk of breast cancer in premenopausal women Cancer Epidemiol Biomarkers Prev 2004 13 1032 1036 15184260

[B14] Kaaks R Berrino F Key T Rinaldi S Dossus L Biessy C Secreto G Amiano P Bingham S Boeing H Bueno de Mesquita H Chang-Claude J Clavel-Chapelon F Fournier A van Gils C Gonzalez C Gurrea A Critselis E Khaw K Krogh V Lahmann P Nagel G Olsen A Onland-Moret N Overvad K Palli D Panico S Peeters P Quirós J Roddam A Serum sex steroids in premenopausal women and breast cancer risk within the European Prospective Investigation into Cancer and Nutrition (EPIC) J Natl Cancer Inst 2005 97 755 765 10.1093/jnci/dji132 15900045

[B15] Tworoger S Missmer S Eliassen AH Spiegelman D Folkerd E Dowsett M Barbieri R Hankinson S The association of plasma DHEA and DHEA sulfate with breast cancer risk in predominantly premenopausal women Cancer Epidemiol Biomarkers Prev 2006 15 967 971 10.1158/1055-9965.EPI-05-0976 16702378

[B16] Eliassen A Missmer S Tworoger S Spiegelman D Barbieri R Dowsett M Hankinson S Endogenous steroid hormone concentrations and risk of breast cancer among premenopausal women J Natl Cancer Inst 2006 98 1406 1415 10.1093/jnci/djj376 17018787

[B17] Dorgan J Stanczyk F Kahle L Brinton L Prospective case-control study of premenopausal serum estradiol and testosterone levels and breast cancer risk Breast Cancer Res 2010 12 R98 10.1186/bcr2779 21087481 PMC3046441

[B18] Gail MH Brinton LA Byar DP Corle DK Green SB Schairer C Mulvihill JJ Projecting individualized probabilities of developing breast cancer for white females who are being examined annually J Natl Cancer Inst 1989 81 1879 1886 10.1093/jnci/81.24.1879 2593165

[B19] U.S. Preventive Services Task Force Screening for breast cancer: U.S. Preventive Services Task Force recommendation statement Ann Intern Med 2009 151 716 726 W-236 19920272 10.7326/0003-4819-151-10-200911170-00008

[B20] American Cancer Society Guidelines for the Early Detection of Cancer http://www.cancer.org/Healthy/FindCancerEarly/CancerScreeningGuidelines/american-cancer-society-guidelines-for-the-early-detection-of-cancer

[B21] U.S. Preventive Services Task Force Chemoprevention of breast cancer: recommendations and rationale Ann Intern Med 2002 137 56 58 12093249 10.7326/0003-4819-137-1-200207020-00016

[B22] Chlebowski R Col N Winer E Collyar D Cummings S Vogel V Burstein H Eisen A Lipkus I Pfister D American Society of Clinical Oncology technology assessment of pharmacologic interventions for breast cancer risk reduction including tamoxifen, raloxifene, and aromatase inhibition J Clin Oncol 2002 20 3328 3343 10.1200/JCO.2002.06.029 12149307

[B23] Armstrong K Quistberg DA Micco E Domchek S Guerra C Armstrong K Quistberg DA Micco E Domchek S Guerra C Prescription of tamoxifen for breast cancer prevention by primary care physicians Arch Intern Med 2006 166 2260 2265 10.1001/archinte.166.20.2260 17101945

[B24] Waters EA Cronin KA Graubard BI Han PK Freedman AN Waters EA Cronin KA Graubard BI Han PK Freedman AN Prevalence of tamoxifen use for breast cancer chemoprevention among U.S. women Cancer Epidemiol Biomarkers Prev 2010 19 443 446 10.1158/1055-9965.EPI-09-0930 20142242 PMC3038785

[B25] Zhou J Ng S Adesanya Famuiya O Anderson K Bondy CA Testosterone inhibits estrogen-induced mammary epithelial proliferation and suppresses estrogen receptor expression FASEB J 2000 14 1725 1730 10.1096/fj.99-0863com 10973921

[B26] Dimitrakakis C Zhou J Wang J Belanger A LaBrie F Cheng C Powell D Bondy C A physiologic role for testosterone in limiting estrogenic stimulation of the breast Menopause 2003 10 292 298 10.1097/01.GME.0000055522.67459.89 12851512

[B27] Wood C Lees C Cline JM Mammary gland and endometrial effects of testosterone in combination with oral estradiol and progesterone Menopause 2009 16 466 476 10.1097/gme.0b013e318191747a 19265727 PMC2755604

[B28] Davis S Moreau M Kroll R Bouchard C Panay N Gass M Braunstein G Hirschberg A Rodenberg C Pack S Koch H Moufarege A Studd J Testosterone for low libido in postmenopausal women not taking estrogen N Engl J Med 2008 359 2005 2017 10.1056/NEJMoa0707302 18987368

[B29] Glaser R York A Dimitrakakis C Beneficial effects of testosterone therapy in women measured by the validated Menopause Rating Scale (MRS) Maturitas 2011 68 355 361 10.1016/j.maturitas.2010.12.001 21177051

[B30] Toniolo PG Pasternack BS Shore RE Sonnenschein EG Koenig KL Rosenberg C Strax P Strax S Endogenous hormones and breast cancer: a prospective cohort study Breast Cancer Res Treat 1991 18 S23 S26 10.1007/BF02633522 1873553

[B31] Kato I Toniolo P Koenig K Kahn A Schymura M Zeleniuch-Jacquotte A Comparison of active and cancer registry-based follow-up for breast cancer in a prospective cohort study Am J Epidemiol 1999 149 372 378 10025481 10.1093/oxfordjournals.aje.a009823

[B32] Rinaldi S Dechaud H Biessy C Morin-Raverot V Toniolo P Zeleniuch-Jacquotte A Akhmedkhanov A Shore RE Secreto G Ciampi A Riboli E Kaaks R Reliability and validity of commercially available, direct radioimmunoassays for measurement of blood androgens and estrogens in postmenopausal women Cancer Epidemiol Biomark Prev 2001 10 757 765 11440961

[B33] Rinaldi S Geay A Dechaud H Biessy C Zeleniuch-Jacquotte A Akhmedkhanov A Shore RE Riboli E Toniolo P Kaaks R Validity of free testosterone and free estradiol determinations in serum samples from postmenopausal women by theoretical calculations Cancer Epidemiol Biomarkers Prev 2002 11 1065 1071 12376508

[B34] Donner A A review of inference procedures for the intraclass correlation coefficient in the one-way random effects model Int Stat Rev 1986 54 67 82 10.2307/1403259

[B35] Rothman M Carlson N Xu M Wang C Swerdloff R Lee P Goh VHH Ridgway EC Wierman M Reexamination of testosterone, dihydrotestosterone, estradiol and estrone levels across the menstrual cycle and in postmenopausal women measured by liquid chromatography-tandem mass spectrometry Steroids 2011 76 177 182 10.1016/j.steroids.2010.10.010 21070796 PMC3005029

[B36] Missmer S Spiegelman D Bertone Johnson E Barbieri R Pollak M Hankinson S Reproducibility of plasma steroid hormones, prolactin, and insulin-like growth factor levels among premenopausal women over a 2- to 3-year period Cancer Epidemiol Biomarkers Prev 2006 15 972 978 10.1158/1055-9965.EPI-05-0848 16702379

[B37] Burger HG Dudley EC Cui J Dennerstein L Hopper JL A prospective longitudinal study of serum testosterone, dehydroepiandrosterone sulfate, and sex hormone-binding globulin levels through the menopause transition J Clin Endocrinol Metab 2000 85 2832 2838 10.1210/jc.85.8.2832 10946891

[B38] Santen RJ Boyd NF Chlebowski RT Cummings S Cuzick J Dowsett M Easton D Forbes JF Key T Hankinson SE Howell A Ingle J Breast Cancer Prevention Collaborative G Critical assessment of new risk factors for breast cancer: considerations for development of an improved risk prediction model Endocr Relat Cancer 2007 14 169 187 10.1677/ERC-06-0045 17639036

[B39] Rockhill B Spiegelman D Byrne C Hunter DJ Colditz GA Validation of the Gail et al. model of breast cancer risk prediction and implications for chemoprevention J Natl Cancer Inst 2001 93 358 366 10.1093/jnci/93.5.358 11238697

[B40] Rosner B Colditz GA Iglehart JD Hankinson SE Risk prediction models with incomplete data with application to prediction of estrogen receptor-positive breast cancer: prospective data from the Nurses' Health Study Breast Cancer Res 2008 10 R55 10.1186/bcr2110 18598349 PMC2575548

[B41] Helzlsouer K Alberg AJ Bush TL Longcope C Gordon GB Comstock GW A prospective study of endogenous hormones and breast cancer Cancer Detect Prev 1994 18 79 85 8025899

[B42] Eliassen AH Missmer S Tworoger S Hankinson S Endogenous steroid hormone concentrations and risk of breast cancer: does the association vary by a woman's predicted breast cancer risk? J Clin Oncol 2006 24 1823 1830 10.1200/JCO.2005.03.7432 16567770

[B43] Smith R Cokkinides V Brooks D Saslow D Brawley O Cancer screening in the United States, 2010: a review of current American Cancer Society guidelines and issues in cancer screening CA Cancer J Clin 2010 60 99 119 10.3322/caac.20063 20228384

[B44] Gail MH Costantino JP Bryant J Croyle R Freedman L Helzlsouer K Vogel V Weighing the risks and benefits of tamoxifen treatment for preventing breast cancer J Natl Cancer Inst 1999 91 1829 1846 10.1093/jnci/91.21.1829 10547390

[B45] Ozanne E Wittenberg E Garber J Weeks J Breast cancer prevention: patient decision making and risk communication in the high risk setting Breast J 2010 16 38 47 10.1111/j.1524-4741.2009.00857.x 19889168

[B46] Stanczyk F Measurement of androgens in women Semin Reprod Med 2006 24 78 85 10.1055/s-2006-939566 16633981

[B47] Blair I Analysis of estrogens in serum and plasma from postmenopausal women: past present, and future Steroids 2010 75 297 306 10.1016/j.steroids.2010.01.012 20109478 PMC2840185

[B48] Sieri S Krogh V Bolelli G Abagnato CA Grioni S Pala V Evangelista A Allemani C Micheli A Tagliabue G Schunemann HJ Menard S Berrino F Muti P Sieri S Krogh V Bolelli G Abagnato CA Grioni S Pala V Evangelista A Allemani C Micheli A Tagliabue G Schunemann HJ Menard S Berrino F Muti P Sex hormone levels, breast cancer risk, and cancer receptor status in postmenopausal women: the ORDET cohort Cancer Epidemiol Biomarkers Prev 2009 18 169 176 10.1158/1055-9965.EPI-08-0808 19124495

[B49] Farhat G Cummings S Chlebowski R Parimi N Cauley J Rohan T Huang A Vitolins M Hubbell FA Manson J Cochrane B Lane D Lee J Sex hormone levels and risks of estrogen receptor-negative and estrogen receptor-positive breast cancers J Natl Cancer Inst 2011 103 562 570 10.1093/jnci/djr031 21330633 PMC3071353

[B50] Key T Endogenous oestrogens and breast cancer risk in premenopausal and postmenopausal women Steroids 2011 76 812 815 10.1016/j.steroids.2011.02.029 21477610

[B51] The Endogenous Hormones Breast Cancer Collaborative Group Body mass index, serum sex hormones, and breast cancer risk in postmonopausal women J Natl Cancer Inst 2003 95 1218 1226 12928347 10.1093/jnci/djg022

[B52] van den Brandt PA Spiegelman D Yaun SS Adami HO Beeson L Folsom AR Fraser G Goldbohm RA Graham S Kushi L Marshall JR Miller AB Rohan T Smith-Warner SA Speizer FE Willett WC Wolk A Hunter DJ Pooled analysis of prospective cohort studies on height, weight, and breast cancer risk Am J Epidemiol 2000 152 514 527 10.1093/aje/152.6.514 10997541

[B53] Tehard B Clavel Chapelon F Several anthropometric measurements and breast cancer risk: results of the E3N cohort study Int J Obes 2006 30 156 163 10.1038/sj.ijo.0803133 PMC1903368 16231021

[B54] Somboonporn W Davis S Testosterone effects on the breast: implications for testosterone therapy for women Endocr Rev 2004 25 374 388 10.1210/er.2003-0016 15180949

[B55] Liao D Dickson R Roles of androgens in the development, growth, and carcinogenesis of the mammary gland J Steroid Biochem Mol Biol 2002 80 175 189 10.1016/S0960-0760(01)00185-6 11897502

[B56] Tiefenbacher K Daxenbichler G The role of androgens in normal and malignant breast tissue Breast Care 2008 3 325 331 20824027 10.1159/000158055 PMC2931104

[B57] Hofling M Hirschberg A Skoog L Tani E Hagerstrom T von Schoultz B Testosterone inhibits estrogen/progestogen-induced breast cell proliferation in postmenopausal women Menopause 2007 14 183 190 10.1097/01.gme.0000232033.92411.51 17108847

[B58] Campagnoli C Colombo P De Aloysio D Gambacciani M Grazioli I Nappi C Serra G Genazzani A Positive effects on cardiovascular and breast metabolic markers of oral estradiol and dydrogesterone in comparison with transdermal estradiol and norethisterone acetate Maturitas 2002 41 299 311 10.1016/S0378-5122(01)00300-0 12034517

[B59] Davis S Papalia M-A Norman R O'Neill S Redelman M Williamson M Stuckey BGA Wlodarczyk J Gard'ner K Humberstone A Safety and efficacy of a testosterone metered-dose transdermal spray for treating decreased sexual satisfaction in premenopausal women: a randomized trial Ann Int Med 2008 148 569 577 18413618 10.7326/0003-4819-148-8-200804150-00001

[B60] Tamimi R Hankinson S Chen W Rosner B Colditz G Combined estrogen and testosterone use and risk of breast cancer in postmenopausal women Arch Int Med 2006 166 1483 1489 10.1001/archinte.166.14.1483 16864758

[B61] Ness R Albano J McTiernan A Cauley J Influence of estrogen plus testosterone supplementation on breast cancer Arch Int Med 2009 169 41 46 10.1001/archinternmed.2008.507 19139322

[B62] Rosner W Auchus R Azziz R Sluss P Raff H Position statement: Utility, limitations, and pitfalls in measuring testosterone: an Endocrine Society position statement J Clin Endocrinol Metab 2007 92 405 413 17090633 10.1210/jc.2006-1864

